# Prognosis of cirrhotic patients admitted to the general ICU

**DOI:** 10.1186/s13613-016-0194-9

**Published:** 2016-10-05

**Authors:** Gaël Piton, Claire Chaignat, Mikhael Giabicani, Jean-Paul Cervoni, Fabienne Tamion, Emmanuel Weiss, Catherine Paugam-Burtz, Gilles Capellier, Vincent Di Martino

**Affiliations:** 1 Intensive Care Unit, Besançon University Hospital, 25030 Besançon, France; 2 Université de Franche Comté, 25000 Besançon, France; 3 Intensive Care Unit, Rouen University Hospital, Rouen, France; 4 Hepatology Unit, Besançon University Hospital, Besançon, France; 5 Intensive Care and Anesthesiology Department, AP-HP, Hôpital Beaujon, Hôpitaux Universitaires Paris Nord Val de Seine, 75018 Paris, France; 6 University Paris Diderot, Sorbonne Paris Cité, 75018 Paris, France

## Abstract

**Background:**

The prognosis of cirrhotic patients admitted to the ICU is considered to be poor but has been mainly reported in liver ICU. We aimed to describe the prognosis of cirrhotic patients admitted to a general ICU, to assess the predictors of mortality in this population, and, finally, to identify a subgroup of patients in whom intensive care escalation might be discussed.

**Results:**

We performed a retrospective monocentric study of all cirrhotic patients consecutively admitted between 2002 and 2014 in a general ICU in a regional university hospital. Two hundred and eighteen cirrhotic patients were admitted to the ICU. The 28-day and 6-month mortality rates were 53 and 74 %, respectively. Among the 115 patients who were discharged from ICU, only eight patients underwent liver transplantation, whereas 48 had no clear contraindication. Multivariable analyses on 28-day mortality identified three independent variables, incorporated into a new three-variable prognostic model as follows: SOFA ≥ 12 (OR 4.2 [2.2–8.0]; 2 points), INR ≥ 2.6 (OR 2.5 [1.3–4.8]; 1 point), and renal replacement therapy (OR 2.3 [1.1–5.1]; 1 point). For a value of the score at 4 (16 % of patients), 28-day and 3-month mortality rates were 91 and 100 %, respectively. An external validation of the score among 149 critically ill cirrhotic patients showed a good accuracy for predicting in-ICU mortality.

**Conclusions:**

Mortality of cirrhotic patients admitted to a general ICU was comparable to that of other studies. A pragmatic score integrating the SOFA score, INR, and the need for extrarenal epuration was strongly associated with mortality. Among the 16 % of patients presenting with score 4 at ICU admission, 100 % died in the 3-month follow-up period. The prognostic evaluation on day 3 remains essential for the majority of patients. However, this score calculable at ICU admission might identify patients in whom the benefit of intensive care escalation should be discussed, in particular when liver transplantation is contraindicated.

**Electronic supplementary material:**

The online version of this article (doi:10.1186/s13613-016-0194-9) contains supplementary material, which is available to authorized users.

## Background

The recent developments in the global management of ICU patients and the improved understanding of cirrhosis physiopathology and complications probably account for the better short-term survival of cirrhotic patients reported in the latest studies [1]. Nonetheless, the prognosis of cirrhotic patients admitted to the intensive care unit (ICU) remains poor. Data from the literature of the last decade describe ICU mortality ranging from 36 to 65 % [2–5] and in-hospital mortality varying from 44 to 86 % [6–8], suggesting that despite the initial control of organ failures before release from ICU, 8–21 % of additional patients die in the short term.

 An early prognostic assessment is crucial for discriminating the good candidates for ICU from those for whom the situation is hopeless despite strong therapeutic interventions. In this perspective, it has been demonstrated that standard prognostic scores used by hepatologists, such as MELD or Child–Pugh, are insufficient because of focusing on the liver without considering other failures and the hepatic state. The ICU general scores, which reflect the state of the main living functions, are more relevant, particularly in the short term [2, 9–11].

Recently, the literature has underlined the relevance of a prognostic assessment deferred to day 3, which seems more reliable than an assessment on admission [2, 5, 12]. However, the application of such deferred prognostic assessment that can only occur in ICU raises two practical issues: The first is the “permissive” access of cirrhotic patients in ICU and the second is the interruption of organ replacement therapy at day 3 when the situation is deemed hopeless.

The primary objective of our study was to describe the short-term and long-term outcome of cirrhotic patients admitted to a non-specialized general ICU. The secondary objectives were, first, to analyze the performance of prognostic markers to predict 28-day mortality and, second, to develop and evaluate a new prognostic score determined at ICU admission that may identify patients with an extremely poor prognosis in whom intensive care escalation should be discussed, in particular if liver transplantation is contraindicated.

## Patients and methods

### Population

This is an observational, monocentric cohort study carried out in the medical ICU of the Besancon University Hospital. All cirrhotic patients (cirrhosis either histologically proven or diagnosis made by hepatologists according to clinical, biological, and ultrasonographic criteria) admitted to our medical ICU between 01.01.2002 and 30.05.2014 were included. When a patient had been admitted to ICU more than once, only the first admission was analyzed.

### Data collection

For each patient, 170 clinical and paraclinical variables were collected from their medical charts. These parameters were known at ICU admission (*n* = 110) and during ICU stay (*n* = 60). Clinical data included socio-demographic characteristics, comorbidities, cause of admission, history of cirrhosis, baseline clinical conditions, and therapeutics during ICU stay. Biological data included 34 biochemical and bacteriological variables, allowing the calculation of 7 prognostic scores at baseline and day 3. The prognostic scores assessed were the Child–Pugh score, MELD score, D’Amico classification, IGS II score, APACHE II score, SOFA score, and CLIF-SOFA score calculated as global scores.

The main assessment criterion was 28-day mortality. The vital status was confirmed by the review of the computer-based medical record of the hospital which complies information from all units (Axigate database). When a patient released hospital alive, if no data were available after this release, the time was censored at the date of hospital release for statistical analyses.

### Statistical analyses

Qualitative variables were expressed by the number and percentages and compared by Fisher’s exact tests. Quantitative variables were expressed by the median and interquartile range (IQR) and compared with Wilcoxon’s rank test. Survival analyses used the Kaplan–Meier model and log-rank tests. Multivariable analyses of predictors of mortality used a logistic regression model for 28-day mortality. Variables included in the multivariable model were chosen according to univariate analyses, their relevance according to the literature, and the likelihood of collinearity based upon clinical judgment. Indeed, we considered that general ICU scores, all evaluating the risk of mortality and being based upon the sequential assessment of organ failures, would be collinear. Similarly, we considered that validated biological variables of liver failure (i.e., bilirubin, prothrombin rate, and international normalized ratio) would be collinear. A multiple logistic regression model was used for the creation of a new prognostic score, based on 28-day mortality, and taking into account variables collected at the time of ICU admission. This score incorporated among the general ICU scores, the markers of liver failure, and the medical interventions, the one of each which had the best prognostic performance. The local score was externally evaluated in a cohort of 149 cirrhotic patients admitted to the ICU of the Rouen University Hospital. Statistical analyses were performed using SAS 9.3.

## Results

### Patient baseline characteristics

In the study period, 7427 admissions were recorded, including 258 (3.5 %) admissions for cirrhosis-related complications. Those 258 admissions corresponded to 218 patients. The baseline characteristics of patients are described in Additional file 1: Table S1. Care and outcome in ICU are described in Additional file 2: Table S2. Briefly, the median age was 59 [51–67], there were 165 men (76 %), and alcoholism was the main cause of cirrhosis (85 %). On admission, the medians of the different prognostic scores were as follows: Child–Pugh = 11 [IQR 9–12], MELD = 28 [18–40], SOFA = 12 [9–15], CLIF-SOFA 14 [11–17], and IGS II = 59 [45–72]. The reasons for admission included: hemodynamic failure (53 %), coma (53 %), respiratory failure (36 %), renal failure (25 %), septic shock (17 %), and gastrointestinal bleeding (13 %). One hundred and four patients (48 %) had more than one reason for ICU admission. During ICU stay, 190 patients needed mechanical ventilation (90 %). Of them, 173 (79 %) had been intubated at ICU admission. One hundred and eighty-seven patients (86 %) received catecholamines and 112 (51 %) needed a renal replacement therapy (22 of them (10 %) a MARS).

### Outcome events

The median duration stay in ICU was 5 days [IQR 2–12]. The median duration of hospitalization was 13 days [4–36]. In-ICU, 28-day, 3-, 6-month, and 1-year mortality rates were 47 % (103/218), 53 % (116/218), 66 % (139/210), 74 % (145/196), and 77 % (147/190), respectively (Additional file 2: Table S2; Additional file 3: Fig. S1).

The outcome of the 115 ICU survivors is described in Additional file 4: Fig. S2. Forty-eight cirrhotic patients were apparently eligible for liver transplantation according to our retrospective review. Conversely, 67 patients were not good candidates because of old age (*n* = 19), alcohol dependence (*n* = 34), possible advanced hepatocellular carcinoma (*n* = 6), or extrahepatic malignancy (*n* = 12). Finally, only eight (17 %) patients underwent liver transplantation after their ICU stay.

### Univariate analysis according to the study period

Renal replacement therapy at ICU admission was performed in 25 and 25 % of the patients before and after 2010 (*p* = 1). Catecholamine use at ICU admission was given in 64 and 77 % of the patients before and after 2010, respectively (*p* = 0.04). Mechanical ventilation was present in 62 and 76 % of the patients before and after 2010, respectively (*p* = 0.03). Liver transplantation during or after ICU stay was performed in five patients in the first study period and in three patients in the second study period. The SOFA score at ICU admission was not significantly different between these two periods (medians of 11 and 12, respectively, *p* = 0.25).

### Univariate analysis of factors associated with 28-day mortality

Results of univariate analysis of factors associated with 28-day mortality are given in Table 1. Factors significantly associated with 28-day mortality included low body temperature, low arterial pressure, admission for severe sepsis or septic shock, high INR, high plasma concentrations of creatinine, lactate, and bilirubin, low pH, PaO2/FiO2, platelet count, low albumin, low prothrombin rate, the need of mechanical ventilation, renal replacement therapy, catecholamines, and high levels of all prognostic scores. The period of admission (before or after 2010) did not have significant impact on mortality (log rank *p* = 0.45).

**Table 1:** Predictive factors of 28-day mortality determined by univariate analysis in 218 cirrhotic patients admitted to the ICU

	28-day survivors (*n* = 102)	28-day non-survivors (*n* = 116)	*p*
Variable			
Age	57 (50–66)	59 (51–68)	0.47
Male sex	75 (74)	90 (78)	0.53
Hepatology transfer	31 (30)	54 (47)	0.02
Diabetes mellitus	36 (35)	24 (21)	0.02
Smoker	88 (86)	89 (77)	0.08
Chronic renal failure	9 (9)	10 (9)	1
Cancer	15 (15)	15 (13)	0.84
Characteristics of cirrhosis			
Alcoholic cause	83 (81)	101 (88)	0.26
Viral cause	14 (14)	18 (16)	0.70
Esophageal varices	72 (71)	86 (75)	0.54
Ascites	63 (62)	86 (74)	0.06
Diagnosis at ICU admission			
Hemodynamic failure	34 (34)	81 (70)	<0.0001
Gastrointestinal bleeding	13 (13)	15 (13)	1
Respiratory failure	43 (42)	36 (31)	0.09
Severe sepsis	18 (18)	42 (36)	0.002
Septic shock	12 (12)	26 (22)	0.048
Renal failure	20 (20)	34 (29)	0.12
Neurological failure	60 (59)	56 (48)	0.14
Cardiorespiratory arrest	2 (2)	16 (14)	0.002
Clinical parameters at ICU admission			
Temperature	37.0 (36.1–37.6)	36.0 (35.0–37.0)	<0.0001
Systolic arterial pressure	110 (100–126)	100 (90–112)	<0.0001
Mean arterial pressure	70 (65–80)	62 (55–68)	<0.0001
Biological parameters at ICU admission			
International normalized ratio	2.1 (1.7–2.8)	3.6 (2.3–6.5)	<0.0001
Blood glucose (mmol/L)	7.4 (6.0–9.5)	6.0 (3.7–8.4)	0.0002
Creatinine (µmol/L)	127 (78–210)	186 (118–293)	0.001
pH	7.37 (7.29–7.44)	7.25 (7.09–7.39)	<0.0001
Lactate (mmol/L)	2.4 (1.7–3.6)	4.7 (2.5–11.3)	<0.0001
PaO_2_/FiO_2_	290 (190–400)	200 (118–348)	0.003
White blood cells (×10^9^/L)	10.5 (7.4–15.5)	12.5 (8.4–19.0)	0.03
Hemoglobin (g/dL)	9.9 (8.8–11.4)	8.8 (7.4–11.0)	0.005
Platelets (×10^9^/L)	113 (79–172)	90 (62–141)	0.005
Bilirubin (µmol/L)	37 (20–90)	112 (34–248)	<0.0001
Albumin (g/L)	26 (22–30)	22 (18–28)	0.002
Prothrombin rate (%)	43 (33–54)	25 (16–39)	<0.0001
Prognostic scores at ICU admission			
Child–Pugh	10 (8–12)	11 (10–13)	<0.0001
MELD	21 (15–29)	35 (25–45)	<0.0001
IGS II	49 (39–60)	68 (57–81)	<0.0001
APACHE II	23 (18–27)	28 (23–34)	<0.0001
SOFA	10 (7–12)	14 (11–17)	<0.0001
CLIF-SOFA	13 (10–15)	16 (13–19)	<0.0001
Prognostic score after ICU admission			
SOFA at day 3	8 (5–10)	16 (12–19)	<0.0001
Clinical feature during ICU stay			
Mechanical invasive ventilation	76 (75)	106 (91)	0.009
Vasopressor therapy	78 (76)	109 (94)	0.0003
Renal replacement therapy	28 (27)	84 (72)	<0.0001
MARS	8 (8)	14 (12)	0.37
Clinical course in ICU			
Documented infection	76 (75)	94 (81)	0.26
Severe sepsis	56 (55)	88 (76)	0.002
Septic shock	35 (34)	63 (54)	0.004
Gastrointestinal bleeding	30 (29)	39 (34)	0.56
Hepatic encephalopathy	64 (63)	57 (49)	0.06

Numbers are *n* (%), median [interquartile range], *MARS* molecular adsorbent recirculating system

### Multivariable analysis of predictors of mortality

We aimed to build a multivariable model including the best general ICU score, the best biological variable of hepatic failure, and the most relevant therapy performed at ICU admission. Among general ICU score (SOFA score, CLIF-SOFA, APACHE II), the SOFA score was the variable which was the most strongly associated with 28-day mortality. Among biological variables of liver failure, INR was the variable which was the most strongly associated with 28-day mortality. Among variables of treatment at the time of ICU admission (i.e., renal replacement therapy, catecholamine use, and mechanical ventilation), RRT was the variable which was the most strongly associated with 28-day mortality. According to these results, we built a multivariable model including the SOFA score, INR, and RRT. For the analysis, the SOFA score and INR were dichotomized at their median value in the cohort, respectively, 12 and 2.6. The results of multiple logistic regressions of variables relating to 28-day mortality are given in Table 2. These three variables were independently associated with 28-day mortality: baseline SOFA score ≥ 12 (OR 4.2 [2.2–8.0]), INR ≥ 2.6 at ICU admission (OR 2.5 [1.3–4.8]), and need for RRT at ICU admission (OR 2.3 [1.1–5.1]).

**Table 2:** Logistic regression analyses of factors associated with 28-day mortality

Variable	Simple logistic regression model		Multiple logistic regression model	
	OR [CI 95 %]	*p*	OR [CI 95 %]	*p*
SOFA score				
<12	1	<0.0001	1	<0.0001
≥12	5.6 [3.1–9.9]		4.2 [2.2–8.0]	
INR				
<2.6	1	<0.0001	1	0.008
≥2.6	4.5 [2.5–8.2]		2.5 [1.3–4.8]	
Renal replacement therapy				
No	1	0.0004	1	0.03
Yes	3.4 [1.7–6.8]		2.3 [1.1–5.1]	

*INR* international normalized ratio, *SOFA* sequential organ failure assessment score

### Creation of a local prognostic score at ICU admission and assessment of its performance

**Fig. 1:**
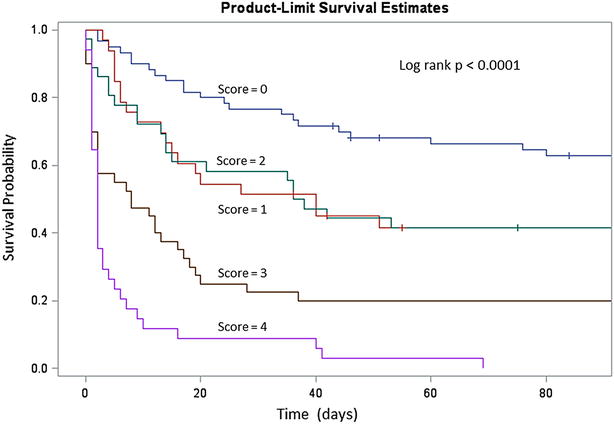
Survival according to our three-variable prognostic score

To give to the clinician a pragmatic, powerful, and easy-to-use tool, we created a prognostic score at ICU admission, called “three-variable prognosis score” to predict 28-day mortality. This score incorporated the three independent mortality risk factors identified by multivariable analysis, each variable of the score being weighted by its odds ratio. Baseline SOFA score ≥ 12 was associated with 2 points; INR ≥ 2.6 at ICU admission was associated with 1 point; and need for RRT at ICU admission was associated with 1 point (Table 3). This score varied from 0 to 4 points and was significantly associated with mortality (log rank *p* < 0.0001, Fig. 1). Log-rank test comparing each strata of the score with each other found that the survival probability for score = 3 and score = 4 was different of each other strata (Additional file 5: Table S3; Additional file 6: Fig. S3). AUC of the score for predicting 28-day mortality was 0.79 [0.73–0.84]. This score calculated at ICU admission was able to discriminate survivors and non-survivors at the times of ICU discharge, 28 days, 3, and 6 months (all *p* < 0.0001). Among patients presenting with score at 4 (16 % of patients), 71 % (24/34) died in the first 3 days after ICU admission and 88 % (30/34) died in the first 10 days. For a value of the score at 4, the prediction of 28-day mortality and 3-month mortality was excellent (positive predictive value of 91 and 100 %, respectively).

**Table 3:** Prognostic score at ICU admission and its performance

Definition (score calculate at admission)	Points
SOFA score	
<12	0
≥12	2
INR	
<2.6	0
≥2.6	1
Indication of renal replacement therapy	
No	0
Yes	1
Total score	(0–4 points)

Local score	28-day mortality (*n* = 203)	3-month mortality (*n* = 195)	6-month mortality (*n* = 181)
Total			
0 point	14/60 (23)	22/56 (39)	26/47 (55)
1 point	16/33 (48)	19/30 (63)	20/30 (67)
2 points	15/36 (42)	21/35 (60)	21/33 (64)
3 points	31/40 (78)	32/40 (80)	32/37 (86)
4 points	31/34 (91)	34/34 (100)	34/34 (100)

*INR* international normalized ratio, *SOFA* sequential organ failure assessment score

### External validation of the prognostic local score

The prognostic value of the local score was evaluated in a cohort of 149 critically ill cirrhotic patients admitted to the ICU of the Rouen University Hospital (Table 4). These patients had a cirrhosis histologically proven or a diagnosis of cirrhosis made by hepatologists according to clinical, biological, and ultrasonographic criteria. The median age was 56 years (50–62), median INR was 2.2 (1.6–3.0), median SOFA score at ICU admission was 11 [7–14], renal replacement therapy was performed in 22 patients (15 %), and in-ICU mortality was 38 %. In-ICU mortality was 11, 25, 62, 66, and 85 % among patients presenting with local score equal to 0, 1, 2, 3, and 4, respectively (Table 4). In-hospital mortality was 100 % for patients with score equal to 4 at ICU admission. Among the 13 patients classified score 4 (i.e., those having SOFA score ≥ 12 and INR ≥ 2.6 and need for RRT), RRT was indicated but not performed in seven patients despite a deep metabolic acidosis, because of lack of time, because of the death occurring in the first hours after admission, or because the clinician estimated that the patient was moribund and RRT was futile. These seven patients died within 24 h of ICU admission.

**Table 4:** External validation of the local score in a cohort of 149 cirrhotic patients admitted to the ICU of the Rouen University Hospital

Local score	In-ICU mortality (*n* = 149)
0 point	7/62 (11)
1 point	6/24 (25)
2 points	13/21 (62)
3 points	19/29 (66)
4 points	11/13 (85)

Numbers are *n* (%)

## Discussion

This monocentric cohort study has evaluated the mortality risk factors in 218 cirrhotic patients consecutively admitted to our general ICU and reports two main results: (1) the prognosis of cirrhotic patients admitted to non-specialized general ICU is comparable to that of other studies, but the long-term prognosis is poor in the absence of liver transplantation; (2) a new prognostic score combining SOFA, INR, and the need for renal replacement therapy improves the prognostic assessment at baseline and could make usefulness the delayed assessment at day 3 in 16 % of patients.

Our study had the merit to collect exhaustive information in a non-selected population from a general ICU in a regional university hospital. Although the proportion of admission for complicated cirrhosis was low during the study period (3.5 %), we were able to report the long-term outcome of almost 200 patients, making our cohort larger than others reported in the majority of published studies on the topic.

In our center, the overall mortality of patients admitted between 2002 and 2014 in our ICU was around 30 %, comparable to that of the literature. In cirrhotic patients, in-ICU mortality was 47 % and was also comparable to that reported in the literature of the last decade, since it ranges from 36 to 65 % [2–5]. Regarding in-ICU and 28-day mortality rates, our data were similar to the 41 % in-ICU and 54 % 28-day mortality rates reported by Das et al. [5], but higher to that reported by Flood et al. [13]. The discrepancies are probably the consequence of the severity of the patients at admission. Interestingly, our results were obtained in a population with a high proportion of mechanical ventilation (90 %). Regarding long-term mortality, our results were close to the 38 % 6-month survival given by Das [5] and the 31 % one-year survival given by Gildea [6].

The result of initial prognostic assessments was consistent with the literature data. In particular, we found a good performance of organ dysfunction scores: A high SOFA score was particularly a good predictor of in-ICU mortality as reported in the study by Levesque et al. [2]. However, we wanted to improve our initial prognostic assessment as much as possible, and this is why we created a new composite score from this series. The statistical analyses allowed us to identify the best score of organ failures, the best marker of liver failure, and the most relevant organ replacement therapy to predict in-ICU mortality. Thus, the three following variables: SOFA ≥ 12, INR ≥ 2.6, and need for renal replacement therapy, were selected and incorporated in our new composite score. This score was efficient for the evaluation of short-term and long-term survival.

Recent literature has emphasized the value of a deferred assessment at day 3, justifying in practice a permissive attitude consisting of undertaking intensive support without restriction but with a re-evaluation by SOFA or modified SOFA score after 72 h [5, 11]. In that context, Ginès et al. [14] indicated that therapeutic limitation could be considered if more than 3 organ failures persist at day 3. Such assessment deferred at day 3 is problematic in everyday practice, when considering the financial and human investment it represents, which may appear disproportionated given the suboptimal efficiency of life supports in these patients. In our center, the implementation of therapeutic limitations remains difficult and inconstant. This is why we wanted to optimize and maximize the predictive value of the initial evaluation to establish a thinner selection of cirrhotic candidates for admission in ICU. Our new prognostic score partially solved this issue. A score equal to 4 at ICU admission was associated with a predictive value of 100 % for 3-month mortality. Similarly, in-hospital mortality was 100 % among patients with score equal to 4 in the validation cohort of the Rouen University Hospital.

Further progress concerns the “hepatology” management of these patients. Surprisingly, although priority is given in France to the sickest patients for organ allocation, only 17 % of patients eligible for transplantation actually underwent liver transplantation after their ICU stay in our series. This paradox is not specific to our center and has been clearly highlighted by a meta-analysis dedicated to the prognosis of more than 2600 cirrhotic patients admitted to ICU and included in 13 international studies: Only 3 % of those patients underwent liver transplantation, whereas 55 % survived to ICU stay (Vincent Di Martino, personal communication). We think an ICU stay should be considered as a major event in the natural history of cirrhosis, able to indicate per se a liver transplantation, as well as other events such as a spontaneous bacterial peritonitis.

Our study acknowledges several limitations: (1) This was a retrospective, monocentric, and observational study. However, an external validation of our local score showed a good accuracy for predicting in-ICU mortality in 149 critically ill cirrhotic patients. In this cohort, there was only 9 % patient with a score equal to 4, suggesting that patients considered as moribund may not be systematically admitted to the ICU. (2) The population of cirrhotic patients who have not been admitted to ICU and the reasons why ICU admission did or not occur are not known. This selection of patients undoubtedly influenced our results in “real life.” However, this criticism applies to all of the series previously published, because the selection procedures regulating access to ICUs of cirrhotic patients were never described. (3) The inclusion period did not allow us to investigate the prognostic impact of new anti-HCV drugs. (4) As in other studies, our statistical analyses and the determination of our prognostic score presupposed that each relevant variable was not linked to the others. However, it is clear that in those patients, organ failures are intricate and also correlated with the degree of liver failure, the reasons for admission, and the therapeutic interventions. In this context, it is probably unrealistic to argue for the identification of entirely “independent” variables. (5) The long-term analyses were conducted on the entire population, which probably overestimates the impact of initial assessments (especially prognostic scores) because the largest proportion of deaths occurs in ICU. It would have been more appropriate to perform a specific analysis restricted to the 115 patients who survived their ICU stay. However, such analysis would probably have been hampered by a lack of power, making difficult the interpretation of negative results. (6) This study reported critically ill patients admitted to the ICU for medical reasons, and thus, perioperative surgical population was not present in this study. (7) Large prospective validation of this score is necessary before raising definitive conclusions, especially on the possibility to avoid an assessment on day 3 for the most seriously ill patients.

In conclusion, the mortality of cirrhotic patients admitted to a general ICU was comparable to that of other studies. The long-term prognosis of critically ill cirrhotic patients remains poor in the absence of liver transplantation. A pragmatic score calculable at ICU admission might identify patients in whom the benefit of intensive care escalation should be discussed, in particular when liver transplantation is contraindicated.

## Additional files

10.1186/s13613-016-0194-9 Clinical and laboratory characteristics of 218 cirrhotic patients at ICU admission.

10.1186/s13613-016-0194-9 Treatment and prognosis of 218 cirrhotic patients admitted to the ICU.

10.1186/s13613-016-0194-9 Estimated probability of survival of 218 cirrhotic patients admitted to the ICU.

10.1186/s13613-016-0194-9 Outcome of the studied population. Of the 115 patients who survived their ICU stay, 48 were theoritically eligible for liver transplantation, but only 8 underwent liver transplantation.

10.1186/s13613-016-0194-9 Survival according to our 3-variables prognostic score comparing score 0, 1 or 2; score 3; and score 4. Log rank test found a significant difference between the three strata of the score.

10.1186/s13613-016-0194-9 Log rank test comparing each strata of the new prognostic score with each other strata.

## Abbreviations

ICU: Intensive care unit
MARS: Molecular absorbent recirculating system
INR: International normalized ratio
SOFA: Sequential organ failure assessment score
RRT: Renal replacement therapy
