# Supplementary material for: Prognosis of cirrhotic patients admitted to the general ICU
Source: Ann Intensive Care. 2016 Oct 5;6:94. doi: 10.1186/s13613-016-0194-9 (PMC5052245; doi:10.1186/s13613-016-0194-9)
Supplement: Supplementary file 1 — 10.1186/s13613-016-0194-9 Clinical and laboratory characteristics of 218 cirrhotic patients at ICU admission. [file 13613_2016_194_MOESM1_ESM.docx]

Supplemental Digital Content-Table 1. Clinical and laboratory characteristics of 218 cirrhotic patients at ICU admission

| **Variable** | **Patients**  **(n = 218)** |
| --- | --- |
| Age | 59 [51-67] |
| Males (%) | 165 (76) |
| Hepatology’s transfer | 85 (39) |
| Diabetes mellitus | 60 (28) |
| Smoker | 177 (81) |
| Chronic renal failure | 19 (9) |
| Hematologic disease | 17 (8) |
| Cancer | 30 (14) |
| **Cirrhosis-related parameters** |  |
| Alcoholic cause Viral cause Metabolic cause | 184 (85)  36 (16)  14 (6) |
| Ongoing alcohol intake | 66 (30) |
| Oesophageal varices | 158 (73) |
| Ascites | 149 (68) |
| History of spontaneous bacterial peritonitis | 41 (19) |
| History of acute alcoholic hepatitis (biopsy) | 46 (21) |
| History of hepatic encephalopathy | 129 (59) |
| History of hepatocellular carcinoma | 16 (7) |
| History of hepatorenal syndrome | 24 (11) |
| History of liver transplantation | 3 (1) |
| History of TIPS | 4 (2) |
| **Diagnosis at ICU admission** |  |
| Hemodynamic failure | 115 (53) |
| Gastrointestinal bleeding | 28 (13) |
| Respiratory failure | 79 (36) |
| Severe sepsis | 60 (28) |
| Septic shock | 38 (17) |
| Renal failure | 54 (25) |
| Neurological failure | 115 (53) |
| Cardiopulmonary arrest | 18 (8) |
| **Clinical parameters** |  |
| Glasgow coma scale | 12 [6-15] |
| Temperature | 36.5 [35.5-37.3] |
| Systolic arterial pressure | 105 [94-120] |
| Mean arterial pressure | 65 [60-75] |
| **Biological parameters at admission** |  |
| International Normalized Ratio | 2.6 [1.9-4.4] |
| Blood glucose (mmol/L) | 6.8 [4.9-9.0] |
| Creatinine (µmol/L) | 158 [93-251] |
| pH | 7.33 [7.18-7.43] |
| Lactate (mmol/L) | 3.1 [1.9-8.0] |
| PaO_2_/FiO_2_ | 250 [150-373] |
| C Reactive Protein (mg/L) | 42 [19-84] |
| White blood cells (x 10^9^/L) | 11.2 [8.0-17.2] |
| Bilirubin (µmol/L) | 52 [26-165] |
| Albumin (g/L) | 24 [19-29] |
| Prothrombin rate (%) | 35 [22-50] |

Data are expressed as n (%) and median (interquartile ranges).

TIPS: Transjugular intrahepatic portosystemic shunt.
