# Supplementary material for: Prognosis of cirrhotic patients admitted to the general ICU
Source: Ann Intensive Care. 2016 Oct 5;6:94. doi: 10.1186/s13613-016-0194-9 (PMC5052245; doi:10.1186/s13613-016-0194-9)
Supplement: Supplementary file 2 — 10.1186/s13613-016-0194-9 Treatment and prognosis of 218 cirrhotic patients admitted to the ICU. [file 13613_2016_194_MOESM2_ESM.docx]

Supplemental Digital Content-Table 2. Treatment and prognosis of 218 cirrhotic patients admitted to the ICU

| **Prognostic scores at ICU admission** | **Patients**  **n = 218** |
| --- | --- |
| Child Pugh | 11 [9-12] |
| MELD | 28 [18-40] |
| Amico | 4 [3-4] |
| IGS II | 59 [45-72] |
| APACHE II | 25 [20-31] |
| SOFA | 12 [9-15] |
| CLIF-SOFA | 14 [11-17] |
|  |  |
| **Prognostic scores after ICU admission** |  |
| SOFA at day-3 | 11 [7-16] |
| Apache II at day-3 | 19 [14-25] |
|  |  |
| **Organ support at ICU admission** |  |
| Mechanical ventilation | 173 (79) |
| Vasopressor therapy | 153 (70) |
| Renal replacement therapy | 55 (25) |
|  |  |
| **Clinical feature during ICU stay** |  |
| Mechanical ventilation | 196 (90) |
| Vasopressor therapy | 187 (86) |
| Renal replacement therapy | 112 (51) |
| Molecular adsorbent recirculating system | 22 (10) |
|  |  |
| **Clinical course in ICU** |  |
| Duration of ICU stay | 5 [2-12] |
| Duration of hospital stay | 13 [4-36] |
| Liver transplantation in ICU | 1 (0.5) |
| Documented infection  Sepsis sévère  Choc septique | 170 (78)  144 (66)  98 (45) |
| Septic shock | 98 (45) |
| Gastrointestinal bleeding | 69 (32) |
| Hepatic encephalopathy | 121 (56) |
| Liver transplantation during or after ICU | 8 (4) |
| Mortality in ICU Mortality at 28 days Mortality at 3 months Mortality at 6 months Mortality at 1 year | 103/218 (47) 116/218 (53) 139/210 (66) 145/196 (74) 147/190 (77) |

Data are expressed as n (%) and median (interquartile ranges)
