# Supplementary figures and images for: Prognosis of cirrhotic patients admitted to the general ICU
Source: Ann Intensive Care. 2016 Oct 5;6:94. doi: 10.1186/s13613-016-0194-9 (PMC5052245; doi:10.1186/s13613-016-0194-9)

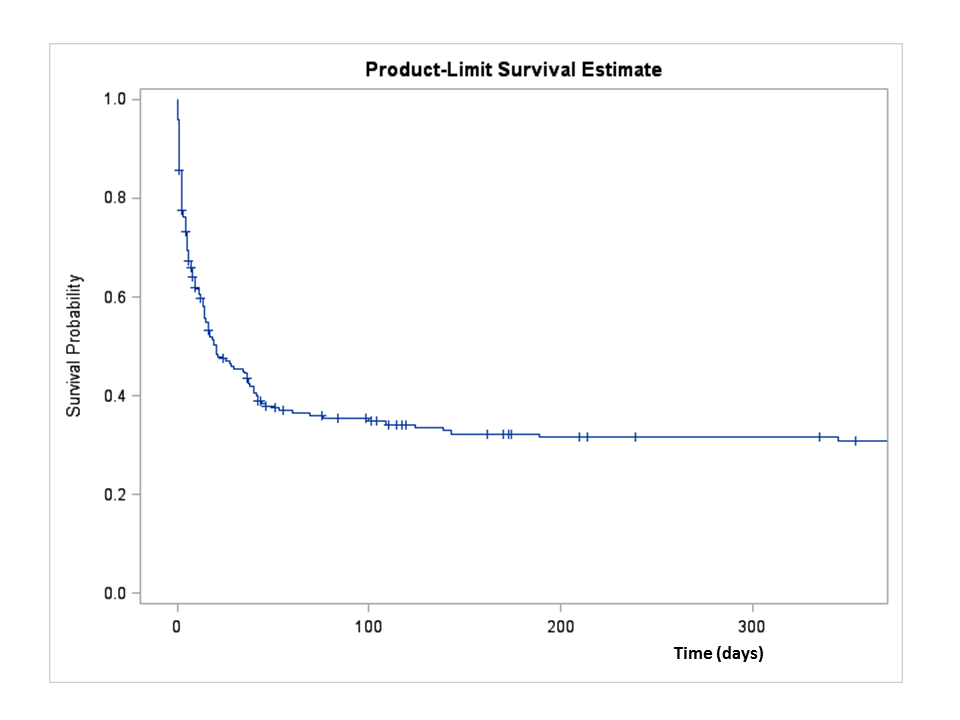

Supplement: Supplementary file 3 — 10.1186/s13613-016-0194-9 Estimated probability of survival of 218 cirrhotic patients admitted to the ICU. [file 13613_2016_194_MOESM3_ESM.tif]

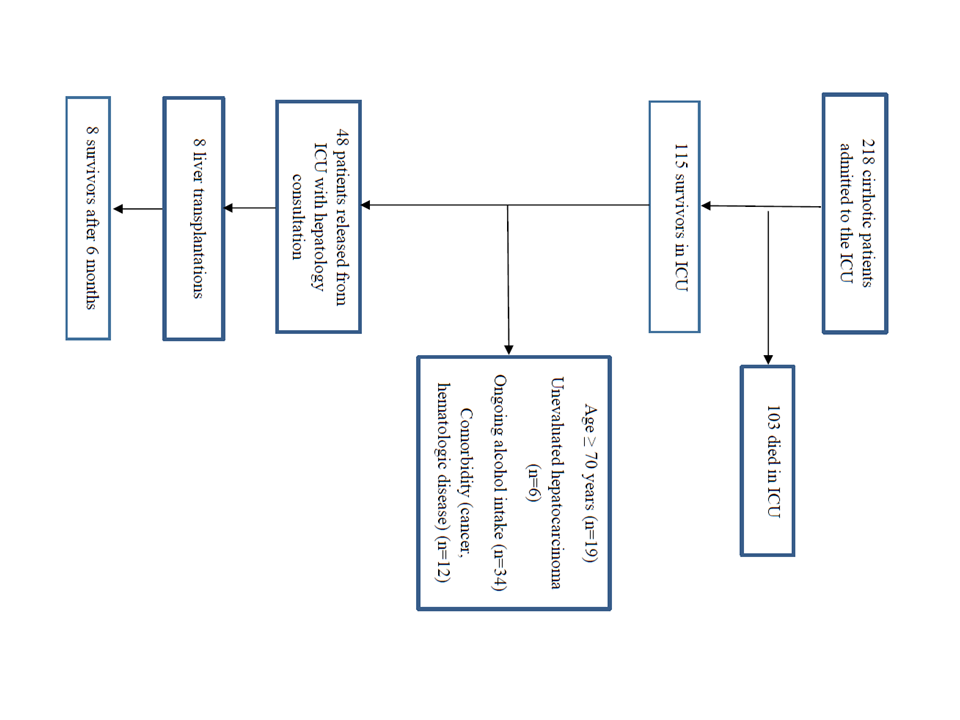

Supplement: Supplementary file 4 — 10.1186/s13613-016-0194-9 Outcome of the studied population. Of the 115 patients who survived their ICU stay, 48 were theoritically eligible for liver transplantation, but only 8 underwent liver transplantation. [file 13613_2016_194_MOESM4_ESM.tif]

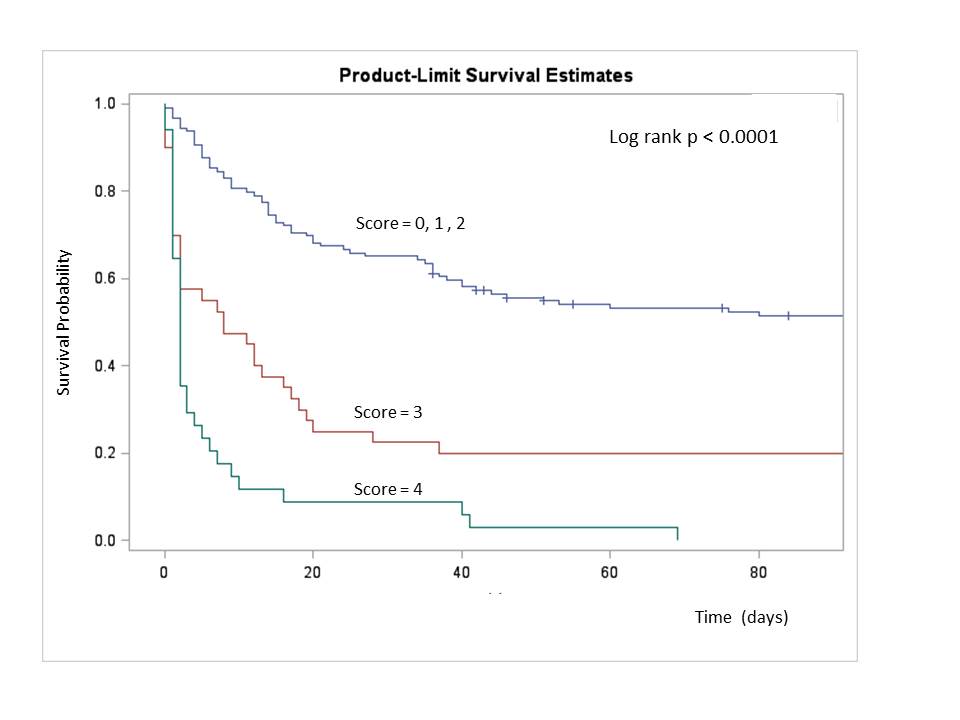

Supplement: Supplementary file 6 — 10.1186/s13613-016-0194-9 Log rank test comparing each strata of the new prognostic score with each other strata. [file 13613_2016_194_MOESM6_ESM.jpg]
