# Supplementary material for: Prognosis of cirrhotic patients admitted to the general ICU
Source: Ann Intensive Care. 2016 Oct 5;6:94. doi: 10.1186/s13613-016-0194-9 (PMC5052245; doi:10.1186/s13613-016-0194-9)
Supplement: Supplementary file 5 — 10.1186/s13613-016-0194-9 Survival according to our 3-variables prognostic score comparing score 0, 1 or 2; score 3; and score 4. Log rank test found a significant difference between the three strata of the score. [file 13613_2016_194_MOESM5_ESM.docx]

Supplemental Digital Content-Table 3. Log rank test comparing each strata of the new prognostic score with each other strata.

| **Log rank p** | **Score = 0** | **Score = 1** | **Score = 2** | **Score = 3** | **Score = 4** |
| --- | --- | --- | --- | --- | --- |
|  |  |  |  |  |  |
| **Score = 0** | - | p = 0.18 | p = 0.39 | p < 0.0001 | p < 0.0001 |
| **Score = 1** | p = 0.18 | - | p = 0.63 | p = 0.02 | p < 0.0001 |
| **Score = 2** | p = 0.39 | p = 0.63 | - | p = 0.007 | p < 0.0001 |
| **Score = 3** | p < 0.0001 | p = 0.02 | p = 0.007 | - | p = 0.004 |
| **Score = 4** | p < 0.0001 | p < 0.0001 | p < 0.0001 | p = 0.004 | - |
